# Tyrosine Kinase Ligand-Receptor Pair Prediction by Using Support Vector Machine

**DOI:** 10.1155/2015/528097

**Published:** 2015-08-11

**Authors:** Masayuki Yarimizu, Cao Wei, Yusuke Komiyama, Kokoro Ueki, Shugo Nakamura, Kazuya Sumikoshi, Tohru Terada, Kentaro Shimizu

**Affiliations:** Department of Biotechnology, The University of Tokyo, 1-1-1 Yayoi, Bunkyo-ku, Tokyo 113-8657, Japan

## Abstract

Receptor tyrosine kinases are essential proteins involved in cellular differentiation and proliferation *in vivo* and are heavily involved in allergic diseases, diabetes, and onset/proliferation of cancerous cells. Identifying the interacting partner of this protein, a growth factor ligand, will provide a deeper understanding of cellular proliferation/differentiation and other cell processes. In this study, we developed a method for predicting tyrosine kinase ligand-receptor pairs from their amino acid sequences. We collected tyrosine kinase ligand-receptor pairs from the Database of Interacting Proteins (DIP) and UniProtKB, filtered them by removing sequence redundancy, and used them as a dataset for machine learning and assessment of predictive performance. Our prediction method is based on support vector machines (SVMs), and we evaluated several input features suitable for tyrosine kinase for machine learning and compared and analyzed the results. Using sequence pattern information and domain information extracted from sequences as input features, we obtained 0.996 of the area under the receiver operating characteristic curve. This accuracy is higher than that obtained from general protein-protein interaction pair predictions.

## 1. Introduction

Tyrosine kinases are enzymes that phosphorylate, particularly tyrosine residues, in proteins. These enzymes are present only in multicellular organisms and play an important role in signal transduction required for cellular differentiation, proliferation, and immune response [1, 2]. Receptor tyrosine kinases are enzymes that are activated by the binding of growth factors and consist of three domains: a transmembrane domain, a ligand-binding extracellular domain, and an intracellular domain that has tyrosine kinase activity [3]. Upon binding of a ligand to the extracellular domain, the receptor tyrosine kinases dimerize and tyrosine kinase domains in the intracellular regions mutually phosphorylate certain tyrosine residues present on each other [3]. This autophosphorylation activates tyrosine kinase and the enzyme gains the capacity to phosphorylate other protein substrates [3].

Receptor tyrosine kinases are essential proteins involved in cellular differentiation and proliferation* in vivo* and are heavily involved in allergic diseases, diabetes, and onset/proliferation of cancerous cells [4]. Identifying the interacting partner of this protein, a growth factor ligand, will improve our understanding of cellular proliferation/differentiation and other cell processes. It may also allow predicting inhibitors of signal transduction, further leading to their practical application in drug design processes, such as the synthesis of an inhibitor that hinders cellular activity [5].

In this study, we developed a new bioinformatics tool to predict the interacting pairs of a receptor tyrosine kinase from amino acid sequences using a machine learning approach based on support vector machine (SVM) [6]. SVM is a supervised machine learning algorithm for solving classification and regression analyses and is known to have high generalization performance. Input data consisted of protein pairs classified into two classes, a binding class and a nonbinding class, which were used for learning and evaluation of the prediction performance of SVM. In SVM prediction, protein information must be extracted as feature values and must be further converted to numerical vectors. The method used for this feature extraction is very important for achieving high prediction accuracy.

Although there are many general protein-protein interaction prediction tools, their performance can be further improved by considering the properties specific to tyrosine kinases. To the best of our knowledge, there have been no such specific prediction tools.

In this study, amino acid sequence pattern information and domain information extracted from sequences were used as feature values. For the sequence pattern information, we used the *k*-mer frequency to convert amino acid sequences into numerical vectors. The *k*-mer frequency [7] is widely used in protein function prediction and considers the appearance frequency of *k* continuous characters within an amino acid sequence as the feature vector. Protein domain is closely related to function and considered to offer effective characteristics for the prediction of protein interaction. For the domain information, both superfamily- and domain-level feature extractions were performed. These features were expected to represent protein function, namely, tyrosine kinase binding facility. We constructed a prediction model based on a single feature, that is, feature using *k*-mer frequency or feature using domain information, and in addition, we attempted to increase prediction accuracy by combining techniques. This paper proposes two combination techniques: combining features of both sequence and domain information to be an input to SVM and combining prediction results obtained by sequence- and domain-based prediction models. We employed C-SVM in LIBSVM library [8] for experiments with SVM.

## 2. Materials and Methods

### 2.1. Dataset

**Figure 1:**
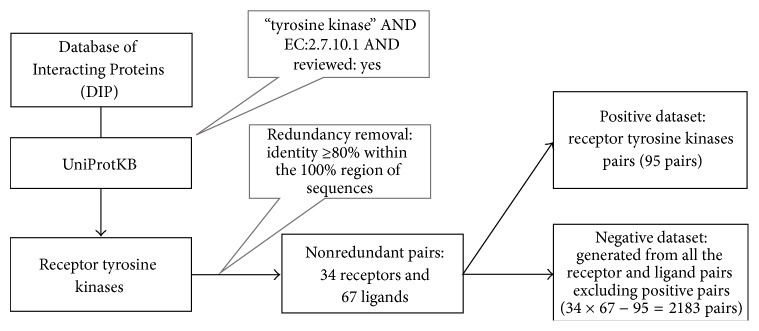
Flow of dataset generation. Data from the Database of Interacting Proteins (DIP) were filtered by selecting only pairs with a protein in the search result of UniProtKB [10] using a keyword and EC number search (“tyrosine kinase” AND EC:2.7.10.1 AND reviewed: yes). To exclude redundancy, clustering was performed against the 174 hits obtained from the interacting pairs of receptor tyrosine kinases and ligands using BLASTclust and one protein was extracted from each cluster. An identity of 80% or above within the 100% region of the amino acid sequence was set as the criteria for BLASTclust. As a result of clustering, 34 receptor tyrosine kinases and 67 ligand proteins were extracted. On the basis of these procedures, 95 pairs were obtained as the final positive data for protein-protein interaction. Negative data (2183) were artificially prepared by excluding the 95 positive data hits from all the combinations of the retrieved receptor tyrosine kinases and their above ligands.

Figure 1 shows the flow of generating our dataset. We collected data from the Database of Interacting Proteins (DIP) [9] in which pairs of interacting proteins are deposited. They were filtered by selecting only the pairs with a protein in the search results of UniProtKB [10] with keyword and EC number search (“tyrosine kinase” AND EC:2.7.10.1 AND reviewed: yes). Furthermore, to exclude redundancy, clustering was performed against the 174 hits obtained from the interacting pairs of receptor tyrosine kinases and ligands using BLASTclust [11] and one protein was extracted from each cluster. An identity of 80% or above within the 100% region of the amino acid sequence was set as the criteria for BLASTclust. As a result of clustering, 34 receptor tyrosine kinases and 67 ligand proteins were extracted. On the basis of the above procedures, 95 pairs were obtained as the final positive data for protein-protein interaction. Negative data (2183) were artificially prepared by excluding the 95 positive data hits from all the combinations of the obtained receptor tyrosine kinases and their abovementioned ligands (34 × 67 − 95 = 2183). Thus, as the sizes of positive data and negative data are imbalanced, we assigned the weights proportional to the sizes using the LIBSVM facilities.

### 2.2. Feature Extraction

We extracted features that were considered to be useful for predicting protein-ligand pairs based on amino acid sequences and domains.

#### 2.2.1. Feature Extraction Based on Amino Acid Sequence

Amino acid sequence is the most basic protein information in terms of functional analysis. For this reason, we performed prediction using amino acid sequence from UniProtKB. We used 1- and 2-mer frequency methods, meaning that 20 dimensions and 20 × 20 = 400 dimensions of feature vectors are acquired, respectively, for each protein. Because we used pairs of proteins as an input, the dimensions of feature vectors given to SVM resulted in 20 × 2 = 40 dimensions and 20 × 20 × 2 = 800 dimensions, respectively.

#### 2.2.2. Feature Extraction Based on Domain Information

**Figure 2:**
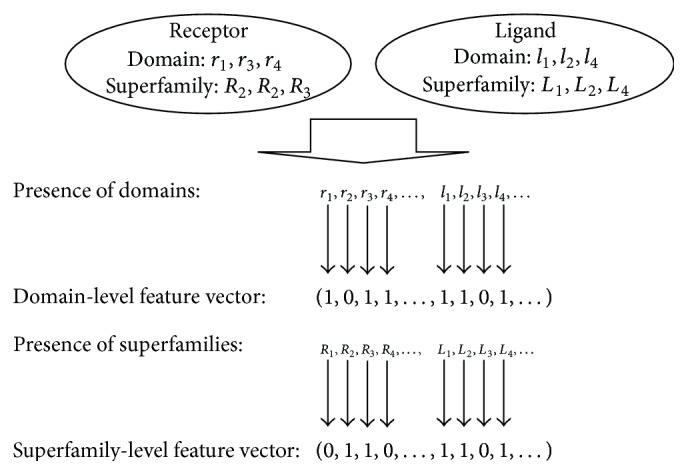
Domain and superfamily encoding. A receptor contains domains *r*_1_, *r*_3_, and *r*_4_. Domains *r*_1_ and *r*_3_ belong to superfamily *R*_2_ and domain *r*_4_ belongs to superfamily *R*_3_. A ligand contains domains *l*_1_, *l*_2_, and *r*_4_, which belong to superfamilies *L*_1_, *L*_2_, and *L*_4_, respectively.

Domain information was acquired from the Conserved Domains Database (CDD) [12]. The *E*-value cutoff parameter for the search was set as 1.0 × 10^−8^. Regarding the receptors, 80 types of domains were extracted by the CDD search and 26 types of superfamilies to which the domains belonged were extracted; in the same way, 98 types of domains and 68 types of superfamilies were extracted for ligands. On the basis of domain and superfamily information, two kinds of feature vectors were generated for numerical representation of receptor-ligand pairs: domain-level and superfamily-level feature vectors.  Figure 2 shows the scheme of the domain and superfamily encoding. In domain-level feature vectors, each vector element corresponds to a domain type. The value of the vector element is 1 or 0 corresponding to whether or not each domain type is present in the protein. The number of dimensions is equal to the number of domain types and the number of dimensions is 178 ( = 80 + 98). In superfamily-level feature vectors, each vector element corresponds to a superfamily type to which the domain type belongs. The value of the vector element is 1 or 0 corresponding to whether or not each superfamily is present in the protein. The number of dimensions is equal to the number of superfamily types and the number of dimensions is 94 ( = 26 + 68).

### 2.3. Integration of Prediction Methods

#### 2.3.1. Combining Feature Vectors

One form of combination applied in our research is to use the combined feature vector, which is created by concatenating each feature vector. This operation was performed so that data characteristics not fully represented by single-feature values could be further detected with other feature values to improve prediction accuracy. The dimension of the combined feature vectors was the sum of the dimensions of the original vectors. The combined feature vectors will hereafter be called “composite vectors.”

#### 2.3.2. Combining Prediction Results

Combining prediction results involves taking a mean value of output values (decision values) from two SVM results using the discriminant function presented in (1). This method is expected not only to improve the prediction accuracy but also to stabilize the accuracy index [13]. Letting the discriminant functions of the prediction models *A* and *B* for data *x* be *f*_*A*_(*x*) and *f*_*B*_(*x*), respectively, the weighted average *f*_comp_(*x*) is given by

(1) $$\begin{array}{c} f_{comp}\left(\;x\;\right)=\frac{af_{A}\left(x\right)+bf_{B}\left(x\right)}{a+b}. \end{array}$$

Here, the weight factors *a* and *b* were given by subtracting 0.5 from area under the receiver operating characteristic curve (AUC) values obtained from prediction models *A* and *B*, that is, the degrees of improvement of accuracy of the predictions over random prediction. The above *f*_comp_(*x*) will hereafter be called “combined results.”

## 3. Results and Discussion

Prediction results were evaluated by 5-fold cross validation. Prediction was performed after determining optimal parameters for SVM. The cross validation was repeated five times, and their average results are shown as the performance.

**Figure 3:**
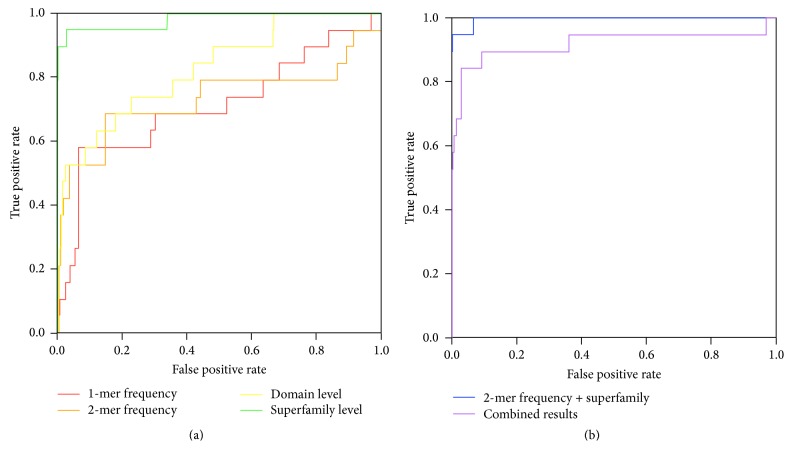
ROC curve of each method. (a) Prediction based on single features. (b) Prediction based on combining methods.

Table 1 shows the prediction results. We have values of true positive (TP), true negative (TN), false positive (FP), and false negative (FN) and performance indexes were calculated by these values: precision *p* is calculated as *p* = TP/(TP + FP), recall *r* is calculated as *r* = TP/(TP + FN), and *F*-measure is calculated as 2*pr*/(*p* + *r*).  Figure 3 shows the receiver operating characteristic (ROC) curve of each prediction method, and AUC is defined as the area under the ROC curve. As shown in the table and the figure, the 2-mer frequency outperformed the 1-mer frequency. The reason is that the 1-mer frequency represents only the amino acid composition and the 2-mer frequency can extract more sequence features than the 1-mer frequency. Prediction methods based on domain information showed higher prediction accuracy than those based on *k*-mer frequencies. Domains are closely related to functions, whereas the *k*-mer frequency can express only the frequencies of *k*-mers of amino acids resulting in rough similarity between two sequences and moreover lacks information about the orders of *k*-mers and binding sites. The prediction method based on the superfamily-level domain achieved the highest prediction accuracy, with precision = 100%, recall = 67.4%, and AUC = 0.974 among the single-feature-based prediction methods. The superfamily-level outperformed the domain-level method because some proteins contain domains not associated with tyrosine kinase specificities. In contrast, superfamily-level information directly represents tyrosine kinase activities without exceptions.

The highest prediction performance was seen when the feature vectors from the 2-mer frequency method and from superfamily-level domains were combined (composite vector), resulting in precision = 98.4%, recall = 69.4%, and AUC = 0.996. Although precision was slightly decreased, recall and AUC were higher than those for the superfamily-level domain. This result indicates the validity of combining the feature vectors obtained from amino acid sequences and domains in the process of predicting receptor tyrosine kinase binding pairs. This result suggests that the two feature vectors complemented each other and led to effective learning.

When the results from the 2-mer frequency method and superfamily-level domain were combined (combined results), the AUC was 0.906, lower by 0.068 than the AUC of the domain before combining. Moreover, precision and recall values decreased. The 0.132 decrease in precision was largely because of a high number of false negative (FP) in the 2-mer frequency method, suggesting that the combination of prediction results is more susceptible to failed prediction than the combination of feature vectors.

For the composite vectors method, we performed an independent test as follows: twenty percent of the positive data (19 pairs) and twenty percent of the negative data (436 pairs) were reserved for testing and the remaining data were used for training. Using only the training data, 5-fold cross validation was performed and optimal SVM parameters were determined based on the average AUC values. With these optimized parameters, independent evaluation was performed for the test data. We repeated the above tests three times and the average performance is as follows: precision = 78.7%, recall = 57.9%, and AUC = 0.858. These results show the practical utility of the composite vectors method.

We also applied our test dataset to a general protein-protein interaction prediction tool, Struct2Net [14], which performs structure-based computational prediction of protein-protein interactions. It successfully predicted 7 of the 95 positive pairs and wrongly predicted 57 of the 2183 negative pairs. This result shows that it is not easy to achieve high accuracy with general protein-protein interaction tools.

We have developed a Web server (http://utprot.net/) for data download.

## 4. Conclusions

In this study, we constructed a tool for predicting interacting pairs of receptor tyrosine kinases and their intra- and extracellular ligands. This tool was intended to provide information to support laboratory experiments. As more data from high-throughput proteomics studies become available and more knowledge is acquired, the reliability of our system's predictions should be improved because SVM performance depends on the features extracted and the quality of the training dataset. We evaluated several input features for SVM, and in particular, by combining domain information and feature vectors of amino acid sequences, we succeeded in obtaining 0.996 of the AUC. We can also use other input features; for example, spatial features may contribute to the improvement of accuracy when the protein structure is known or a highly accurate model is available. Although our domain information is obtained from amino acid sequences, it seems to contain some spatial features and our tool achieved good performance. Moreover, functional information, such as GO terms, gene expression, and metabolic pathways, will further improve the performance. Although it is difficult to select effective features, some prediction tools use novel feature selection methods [15–17]. Our tool may incorporate such feature selection methods in the future. Our method can be applied to other types of protein-protein interactions. Domain- or superfamily-based features and their combination with sequence pattern information will be useful for prediction of specific types of interactions or functions. We are also developing a SVM-based prediction tool for binding affinity of tyrosine kinase ligand-receptor pairs.

## Figures and Tables

**Table 1:** Prediction results of each method.

Method	Precision	Recall	*F*-measure	AUC	TP	FP	TN	FN
1-mer frequency	—	—	—	0.638	0	0	2183	95
2-mer frequency	0.178	0.442	0.253	0.713	42	191	1992	53
Domain level	0.387	0.337	0.357	0.801	32	48	2135	63
Superfamily level	1.0	0.674	0.802	0.974	64	0	2183	31
Composite vector (2-mer frequency + superfamily level)	0.984	0.694	0.812	0.996	66	1	2182	29
Combined results (2-mer frequency + superfamily level)	0.868	0.611	0.712	0.906	58	10	2173	37

Each column in the table describes a single method. “Composite vector” describes the prediction based on the combination of feature vectors from 2-mer frequency method and domain, and “combined results” denotes the combination of the predicted results from 2-mer frequency method and domain (superfamily). TP, FP, TN, and FN: true positive, false positive, true negative, and false negative, respectively.
